# Prevalence of dental caries and associated factors among primary school children in Ethiopia: systematic review and meta-analysis

**DOI:** 10.1186/s12903-024-04555-5

**Published:** 2024-07-10

**Authors:** Amalku Nigussie Yirsaw, Eyob Ketema Bogale, Mitiku Tefera, Mahider Awoke Belay, Ayenew Takele Alemu, Solomon Ketema Bogale, Eyob Getachew, Getnet Alemu Andarge, Kedir Seid, Gebeyehu Lakew

**Affiliations:** 1 Health Promotion and Communication Department, School of public health, College of medicine and health sciences, Gondar University, Gondar, Ethiopia; 2 Health Promotion and Behavioral science department, school of public health, College of medicine and health science, Bahir Dar University, Bahir Dar, Ethiopia; 3 Department of Midwifery, School of Nursing and Midwifery, Debre Birhan University, Asrat Woldeyes Health Science Campus, Debre Birhan, Ethiopia; 4 Department of Public Health, College of Medicine and Health Science, Injibara University, Injibara, Ethiopia; 5 Department of Nutrition, Antsokiya Gemza wereda Health Office, Mekoy, MekoyNorth East Ethiopia; 6 Bati Primary Hospital, Oromo Special Zone, Bati, North Central Ethiopia

**Keywords:** Dental caries, Dental plaque, Primary school children, Ethiopia

## Abstract

**Background:**

Dental caries (decay or cavities) is the breakdown of teeth as a result of bacteria. Dental caries is one of the most preventable oral health problems and the most common chronic disease in primary school children. Poor dental and oral health affects the quality of children’s lives.

**Objectives:**

The study aimed to synthesize the existing literature on the prevalence and associated factors of dental caries among primary school children in Ethiopia in 2024.

**Methodology:**

Studies were searched through the search engines of Google Scholar, PubMed, Scopus, MEDLINE, and the Cochrane Library. Searching was made using keywords and MeSH terms for dental caries, dental plaque, primary school children, and Ethiopia. Heterogeneity was assessed using the Cochran Q test and I2 statistics. A random-effects model with a 95% confidence interval was used for prevalence and odds ratio estimations.

**Result:**

The result of seven studies disclosed that the overall prevalence of dental caries in primary school children in Ethiopia was 35% (26–45%). high intake of sweets (OR = 2.71,95%CI:1.968–3.451), a poor habit of tooth cleaning (OR = 2.46; 95% CI: 2.761–5.045), Grade level 1–4(OR = 2.46; 95% CI: 1.523–3.397), having a history of toothache(OR = 2.99; 95% CI: 2.679–3.314), absence of toothpaste use(OR = 1.42; 95% CI: -1.278-4.109), reduction of the previous year’s academic score(OR = 5.51; 95% CI: 1.952–9.066), had a significant microbial load(OR = 3.82, CI: 3.439–4.192) and have acid bacillary pH on their teeth(OR = 2.42, CI: 1.494–3.335) were independent variables associated with dental carries among primary school children.

**Conclusion:**

The overall prevalence of dental caries among primary school children in Ethiopia is 35%, ranging from 26 to 45%. However, variations in prevalence rates are observed based on sampling techniques. Studies using simple random sampling report a higher prevalence rate of 42%, while those employing multi-stage random sampling and systematic random sampling show lower rates of 30% and 35%, respectively. This indicates that the choice of sampling technique can impact reported prevalence rates, with simple random sampling yielding higher estimates compared to other methods.

## Background

Dental caries, commonly known as tooth decay, ranks among the most widespread chronic diseases globally, affecting individuals throughout their lives. This condition arises from a complex and prolonged interaction between acid-producing bacteria and fermentable carbohydrates, along with various host factors like teeth and saliva. Caries can develop on both the crowns and roots of teeth and can manifest as severe tooth decay in early childhood, impacting the primary teeth of infants and toddlers. The risk factors for dental caries encompass physical, biological, environmental, behavioral, and lifestyle elements, such as a high number of cariogenic bacteria, insufficient salivary flow, lack of adequate fluoride exposure, poor oral hygiene, improper infant feeding practices, and poverty [1].

According to the World Health Organization (WHO), dental caries occurs when the enamel of the tooth is damaged by acids produced by bacteria acting on sugar [2]. It is recognized as a preventable oral health issue and is prevalent globally, particularly affecting children [3, 4]. Approximately 3.5 billion people worldwide suffer from oral diseases, with around 2.4 billion individuals having dental caries in their permanent teeth [5, 6]. Hundreds of millions of children lose primary teeth due to this condition [5]. Lack of health education and preventive measures contribute to its high prevalence and subsequent negative impact on children’s health [7]. In the United States, dental caries are more common than asthma among children [6]. Similarly, England sees a significant number of children admitted to hospitals for teeth removal due to this issue [8].

The incidence of dental caries is rapidly escalating in low- and middle-income countries, especially impacting children residing in underprivileged communities [9, 10]. In Africa, the prevalence of dental caries varies from country to country 78% Eritrea [11], 64%Timor-leste [12], 68.8% São Tomé Island [13], 24.1% Nigeria [14], 78% Tripoli Libya [15], 20% Tunisea [16], 49.7% Ghana [17] and 37.5%,43.3% in Kenya [18, 19], and also Studies conducted in Ethiopia reveal rates of 71.3% community survey in Ethiopia [20], 21.8% in Bahir Dar [21], 47.4% in Addis Ababa [22], 41.5% in Gondar Town [23], and 48.5% in Finote Selam [24]. This condition significantly affects children’s development and their ability to engage in daily activities.

Fluoride exposure, quantity and quality of saliva, and socioeconomic status are crucial factors causing dental caries., along with inadequate tooth-brushing practices, poor oral hygiene, and limited awareness about dental caries, exacerbate the incidence of dental decay [9]. Conversely, regular tooth brushing is associated with a reduced risk of developing dental caries [8, 24].

Globally, a substantial proportion of school children suffer from dental caries, impacting their physical and psychological well-being. Children with dental caries may experience difficulties in sleeping, eating, engaging in education and social activities, affecting their self-esteem and social development [2, 25, 26]. Furthermore, dental caries impose a significant financial burden on parents, particularly in high-income countries where dental treatment is costly. The burden of oral health issues extends beyond individual suffering, with implications for general health, including increased risks of pneumonia, diabetes complications, and infective arthritis [27].

Promoting oral health, including interventions to address dental caries, is essential, and schools can play a crucial role in this regard by implementing comprehensive healthcare programs. However, in countries like Ethiopia, oral health concerns often receive inadequate attention from the government. Therefore, understanding the prevalence and risk factors associated with dental caries can inform targeted interventions aimed at reducing its impact [28].

## Methods

### Study design and search strategy

A systematic search was conducted in several databases including Google Scholar, Scopus, PubMed, and MEDLINE. The search was limited to English-language papers published from the beginning of January 2024 to the end of January 2024. The search utilized specific key terms related to dental caries, tooth decay, or dental plaque, as well as Ethiopia. Additionally, the reference lists of relevant studies were also examined. The search strategy incorporated a combination of keywords to address the main research questions. The strategy included predefined search terms to ensure a comprehensive search, encompassing both text fields within records and Medical Subject Headings (MeSH terms) to expand the search. The specific MeSH terms used for Scopus were: [29] and Ethiopia.

### Study selection and eligibility criteria

The study selection process adhered to the Preferred Reporting Items for Systematic Review and Meta-Analysis (PRISMA) guidelines. Initially, the database search results were combined, and duplicate studies were removed using Endnote software as well as manual screening. Following the removal of duplicates, the titles and abstracts of the remaining studies were screened to exclude those that were irrelevant to the research question and did not align with the study’s outcomes of interest. Full-text studies that met the inclusion criteria were further evaluated. Two authors independently screened the studies using the eligibility criteria and checked for consistency. In cases where there were discrepancies between the authors’ assessments, they were resolved through discussion and consensus. This systematic review and meta-analysis have been registered with PROSPERO, and it has received a registration ID - CRD42024520570.

### Data extraction process

Two investigators (ANY and GL) independently extracted data from the selected studies, including the first author’s name, country, age, study design, sample size, and the prevalence of dental caries or decayed teeth. A standardized data extraction form created in Microsoft Excel version 2019 was used to collect the relevant data. The pooled prevalence of dental caries was also extracted. In cases where there was disagreement between the two reviewers, it was resolved through discussion and consensus.

### Outcome of interest

The outcome variable for this systematic meta-analysis was dental caries among primary school children. According to the World Health Organization (WHO), Dental caries results when plaque forms on the surface of a tooth and converts the free sugars (all sugars added to foods by the manufacturer, cook, or consumer, plus sugars naturally present in honey, syrups and fruit juices) contained in foods and drinks into acids that destroy the tooth over time.

### Study quality and risk of bias

Two independent authors used the Hoy 2012 tool, which consists of ten criteria [30]. to assess the risk of bias in the selected studies. These criteria cover various aspects such as population representation, participant selection methods, non-response bias, data collection procedures, case definition acceptability, reliability and validity of study tools, data collection mode, prevalence period length, and appropriateness of numerator and denominator. Each criterion was evaluated as either low or high risk of bias, and the overall risk of bias for each study was determined based on the total score of high-risk items. The studies were categorized as having low (≤ 2), moderate (3–4), or high (≥ 5) risk of bias.

The GRADE tool (Grading of Recommendations Assessment, Development, and Evaluation) was employed to assess the certainty of evidence for the outcome. The GRADE quality evaluation tool initially considers observational studies as having a low quality of evidence, which can be downgraded further to very low based on factors such as risk of bias, inconsistency, indirectness, imprecision, and publication bias. However, there is an option for upgrading if no other limitations are identified within these factors. Assessments were conducted for five primary domains: risk of bias, consistency, directness, precision, and publication bias. The overall quality of evidence was also evaluated. The study design served as the starting point, and a one-step downgrade was applied for each domain that was not met [29]. also, the quality of the studies was assessed using the Joanna Briggs Institute (JBI) critical appraisal checklist [31, 32]. The reviewers (ANY and EKB) followed a blinded review approach based on a protocol to assess the quality of the original articles. Studies with a score of 5 or more on the JBI criteria were considered to have good quality and were included in the review. Any discrepancies in the quality assessment were resolved through consultation with the discussion.

### Statistical analysis and synthesis

The extracted data from the primary studies, formatted in Microsoft Excel version 19, were imported into the STATA version 17 statistical software for meta-analysis. A random-effects meta-analysis of dental caries was conducted using the DerSimonian and Laird method to account for variability. In cases where standard error (SE) was not provided in the studies, it was calculated in Microsoft Excel. The calculated SE and prevalence data from each study were then used in STATA to calculate the pooled prevalence rate with a 95% confidence interval.

Publication bias was assessed using a funnel plot and visual assessment of its asymmetry. Egger’s and Begg’s tests were also performed at a significance level of 5% to detect publication bias based on the distribution of studies and a *p*-value less than 0.05. The heterogeneity among studies was evaluated using Cochran’s Q test (a *p*-value less than 0.1 indicated significant heterogeneity) and the inverse variance (I^2^) test statistics, which quantified the percentage of total variation in the study estimate due to heterogeneity. An I^2^ value of 75% or higher indicated high heterogeneity, and a *p*-value less than 0.05 indicated statistically significant heterogeneity.

Meta-regression was conducted to explore the potential sources of heterogeneity. The results were presented on a forest plot, displaying the point prevalence with a 95% confidence interval. The size of each box on the forest plot represented the weight of the study. Adjusted odds ratios were used to assess possible associated factors.

Sensitivity analysis was conducted to examine the influence of each study on the pooled effect size. Four investigators independently performed the statistical analysis, and the results were crosschecked for consistency.

## Result

### Characteristics of the studies

**Fig. 1:**
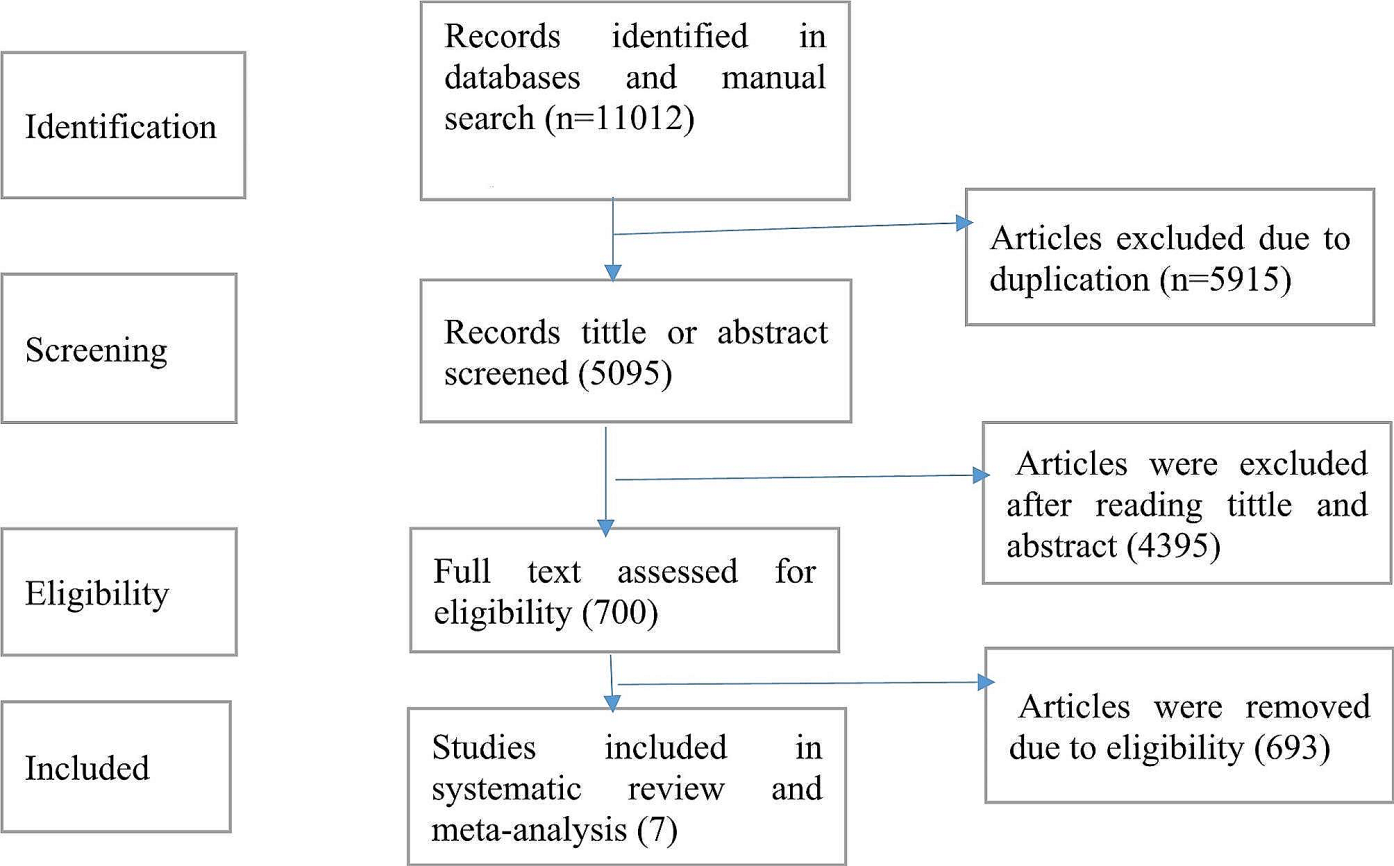
PRISMA flow chart diagram describing the selection of studies for systematic review and meta-analysis on the prevalence of dental caries among primary school children in Ethiopia

A comprehensive search across various databases yielded a total of 11,012 published studies. After removing duplicates, 5,915 studies were excluded. An additional 4,395 studies were excluded based on the relevance of their titles and abstracts to the study’s aim, as determined by the inclusion and exclusion criteria. The remaining 700 full-text articles were thoroughly evaluated for eligibility. Ultimately, 7 studies that met the inclusion criteria were included in the systematic review and meta-analysis (Fig. 1). It is important to note that all seven studies included in the systematic review and meta-analysis were cross-sectional.

**Fig. 2:**
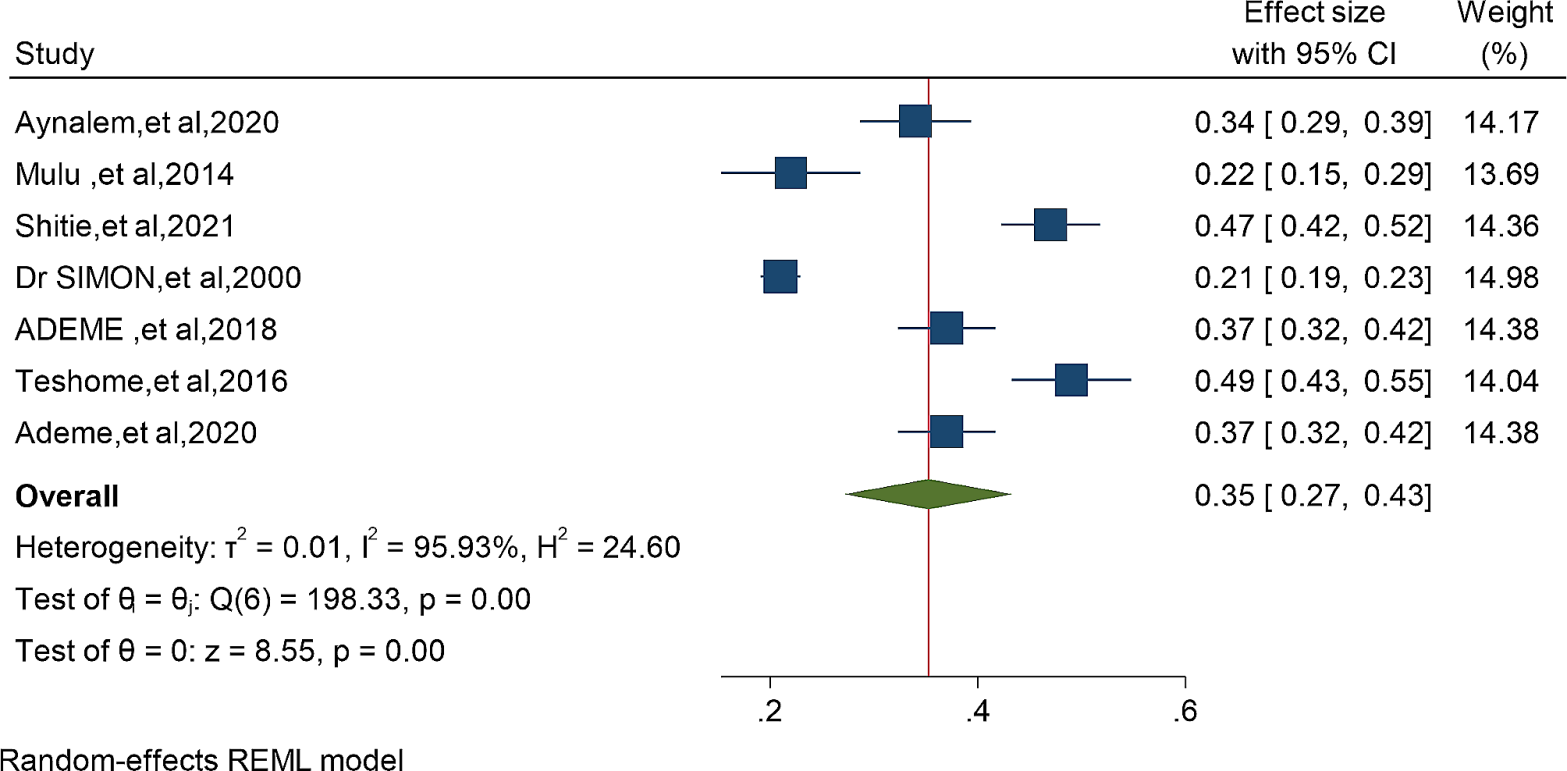
Forest plots of seven studies on the prevalence of dental caries and associated factors among primary school children in Ethiopia: 2024

**Fig. 3:**
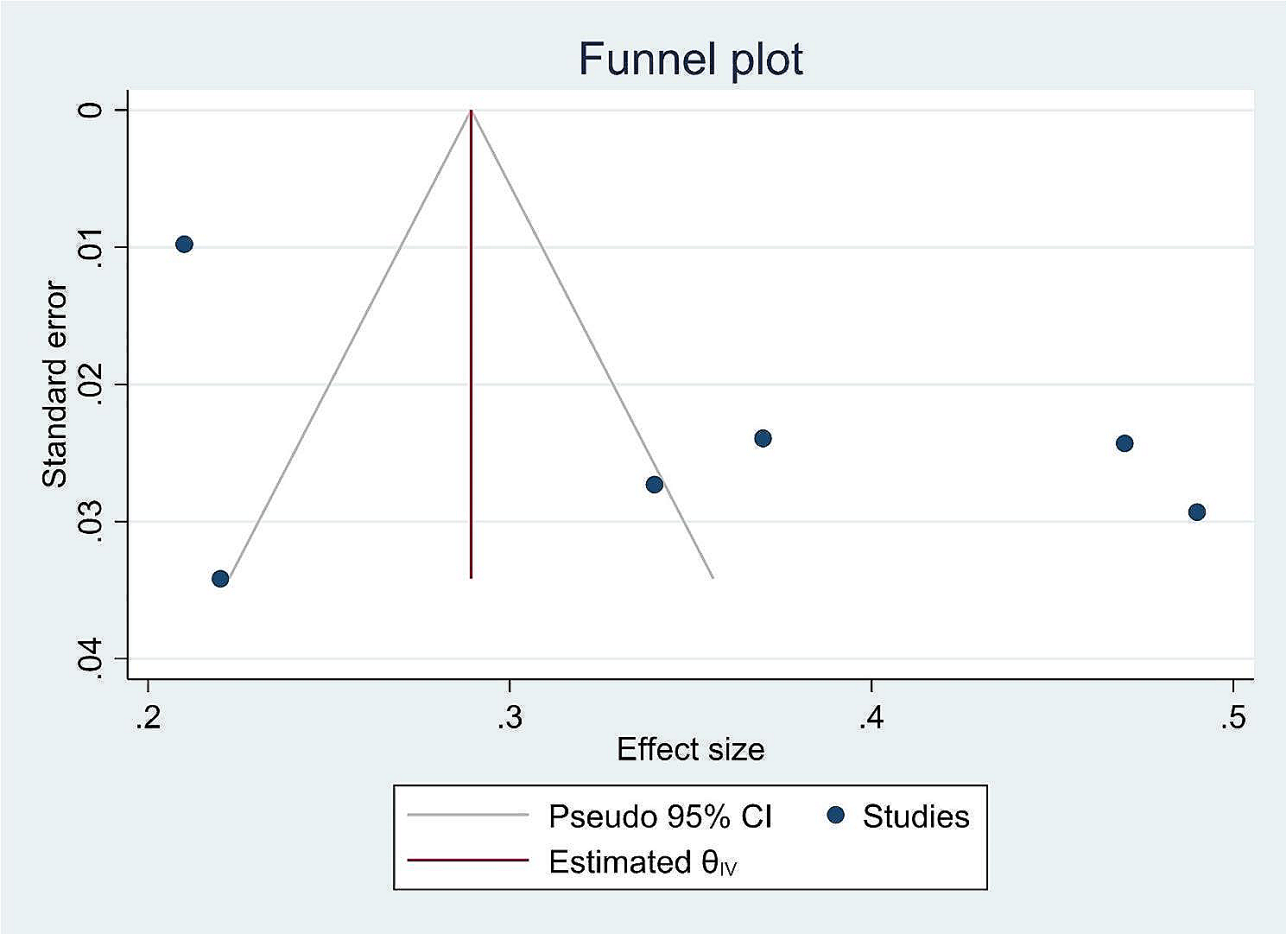
A Funnel plot of studies conducted on the prevalence of dental caries and associated factors among primary school children in Ethiopia: 2024

**Fig. 4:**
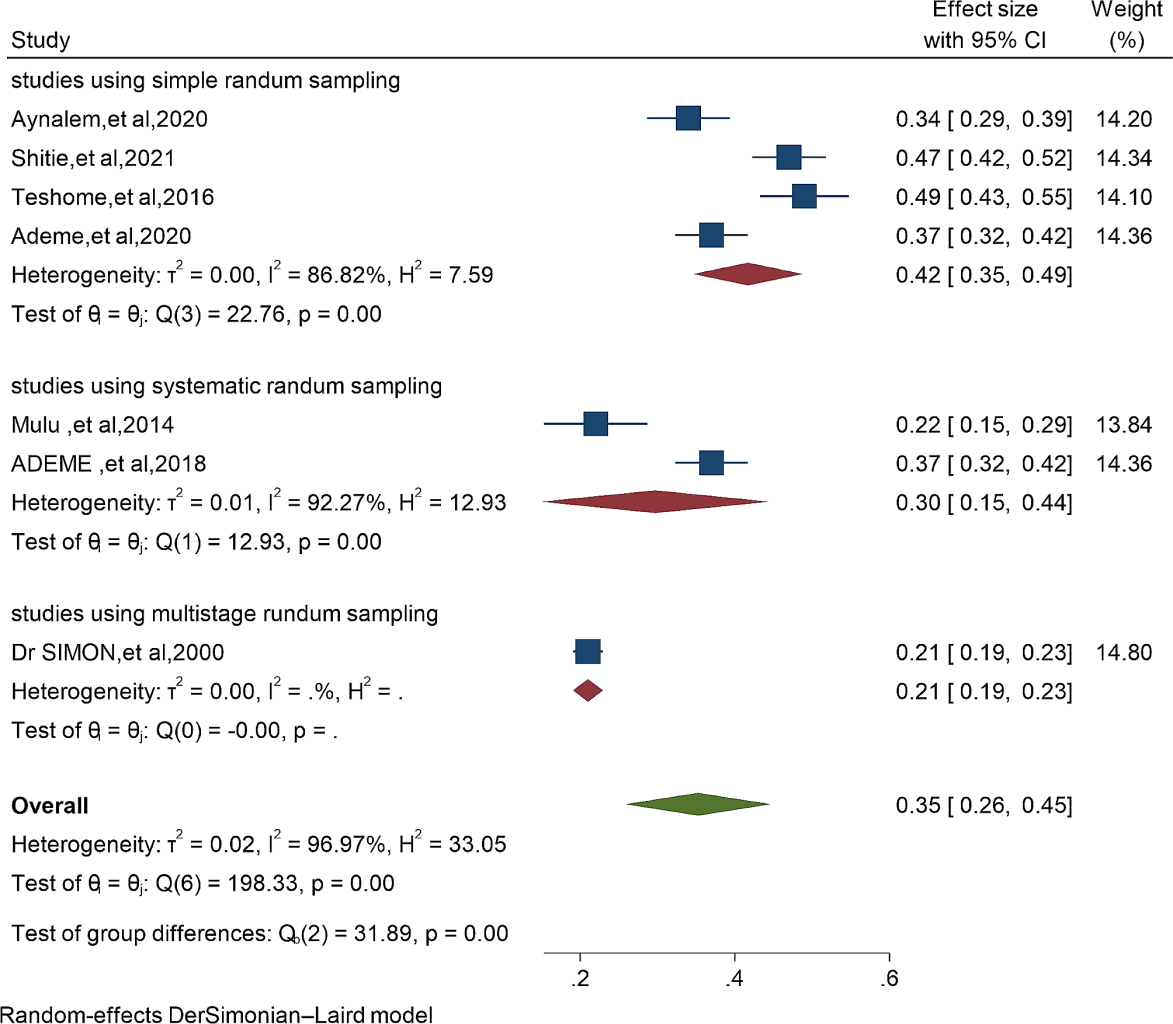
Sub-group analysis based on sampling technique of study on the prevalence of dental caries in Ethiopia, 2024

The risk of bias assessment for the four individual articles included in the systematic review and meta-analysis was conducted using the Hoy 2012 tool, which consists of ten specific criteria as described in the methodology section. Out of the four studies, four (57%) were determined to have a low risk of bias, while the remaining three studies (43%) were classified as having a moderate risk of bias. These seven studies, which investigated the prevalence of dental caries in primary school children, exhibited significant heterogeneity, as indicated by the Cochrane Q test (*p* = 0.00) and I^2^ test (95.93%). Consequently, a random-effects model was employed to account for this heterogeneity (Fig. 2) Even though the Begg rank correlation statistics (*p* = 0.4524) do not show publication bias. the Egger weighted regression statistics for studies on dental caries prevalence (*P* = 0.0440) revealed evidence suggesting the presence of publication bias, and The funnel plot has also an asymmetry by visual inspection also shows there is a sign of publication bias (Fig. 3). To address the observed heterogeneity, a subgroup analysis was conducted based on the sampling technique (Fig. 4) To treat the potential impact of publication bias, a nonparametric trim-and-fill analysis was performed. However, no imputed studies were identified through this analysis.

### Prevalence of dental caries among primary school children in Ethiopia

This systematic review and meta-analysis included seven studies conducted between 2000 and 2022 in Ethiopia, focusing on the prevalence of dental caries among primary school children. The study conducted in 2000 in Addis Ababa reported a prevalence of 21% for dental caries in this population. Similarly, the study conducted in 2014 in Bahir Dar found a prevalence of 22%. In 2016, a study conducted in Finote Selam reported a higher prevalence of 49% among primary school children. Another study conducted in 2018 in Harer reported an even higher prevalence of 63%. In 2020, the study conducted in Debre Birhane observed a prevalence of dental caries of 34% among primary school children, while in Harer Town, it was 37%. The most recent study conducted in 2021 in North Showa reported a prevalence of 47% for dental caries among primary school children. (Table 1)

**Table 1:** Characteristics of the seven studies included in systematic review and meta-analysis

Author	Survey year	Place of the study	Study setting	Sample size	Sampling procedure	Prevalence of dental caries	JBI score
Aynalem	2022	Debrebirhane	Cross-sectional	301	Simple random sampling	34%	8
Mulu	2014	Bahir dar	cross-sectional	147	Systematic random sampling	22%	7
Shitie	2021	North shiwa	cross-sectional	422	Simple random sampling	47%	9
Dr SIMON	2000	Addis Abeba	cross-sectional	1736	Multi-stage random sampling	21%	8
ADEME	2018	Harer	cross-sectional	407	Systematic random sampling	37%	7
Teshome	2016	Finote Selam	cross-sectional	291	Simple random sampling	49%	8
Ademe	2020	Harer town	cross-sectional	407	Simple random sampling	37%	7

The pooled prevalence of dental caries among primary school children in Ethiopia was 35% (95%CI: 27- 43%) (Fig. 2) Furthermore, subgroup analysis based on the sampling technique of the study showed that the prevalence of dental caries in primary school children was significantly higher (42% ) in studies using simple random sampling compared to the lowest prevalence (30% and 35%) in studies using multi-stage random sampling and systematic random sampling respectively (Fig. 4)

### Meta-regression and sensitivity analysis

#### Meta-regression

Meta-regression was performed with the place of study considered as covariates, employing a random-effects model. The outcome indicated the absence of heterogeneity based on the sampling technique of the study (*p* = 0.002) (Table 2).

**Table 2:** Meta-regressions of dental caries by sampling technique of study of included studies in Ethiopia, 2024

Covariate	β (95% CI)	*p*-value
Sampling technique	-0.1063003 (-0.1742799, -0.0383207)	0.002

#### Sensitivity analysis

**Fig. 5:**
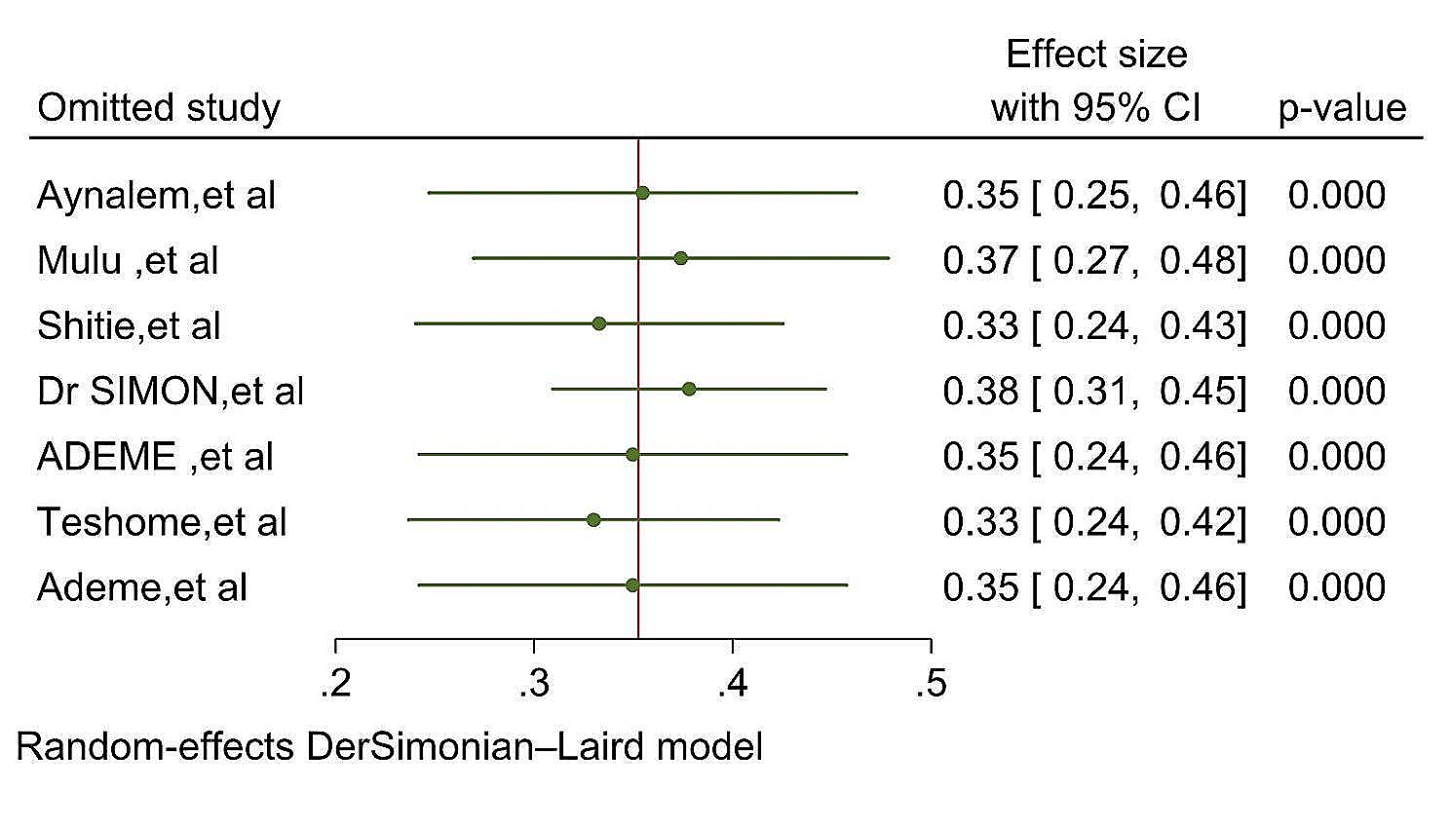
Leave-one-out sensitivity analysis of the prevalence of dental caries in primary school children in Ethiopia, 2024

To assess the influence of individual studies on the overall pooled prevalence of dental caries, a sensitivity analysis was conducted using the leave-one-out method. This involved excluding each study in turn and examining the impact on the estimated prevalence. The results of the sensitivity analysis revealed that the estimated prevalence obtained when excluding each study remained within the confidence interval of the pooled prevalence. Consequently, none of the included studies had a significant effect on the overall pooled estimated below (Table 3 and Fig. 5)

**Table 3:** Sensitivity analysis on prevalence of dental caries in Ethiopia, 2024

Study	Effect size	[95% conf. interval]	*p*-value
Aynalem, et al, 2020	0.355	0.247 0.462	0
Mulu, et al, 2014	0.374	0.269 0.478	0
Shitie, et al, 2021	0.333	0.240 0.425	0
SIMON et al, 2020	0.378	0.309 0.447	0
ADEME, et al, 2020	0.35	0.242 0.457	0
Teshome, et al, 2016	0.33	0.237 0.423	0
Ademe, et al, 2020	0.35	0.242 0.457	0
theta |	0.353	0.259 0.446	0

### Factors associated with dental caries among primary school children in Ethiopia

Before performing the pooled analysis of associated factors, eight factors were identified as being associated with dental caries in primary school children.: high intake of sweets [24, 33–36], food particles on their teeth [33], dental plague [21] sex [24, 27], lack of parent insistence [27], grade level 1–4 [21, 35, 36], a poor habit of tooth cleaning [21, 24, 27, 34–36], toothache [21, 35, 36], reduction of previous year academic year score [35, 36], absence of toothpaste use [35, 36], a significant microbial load of slivery lacto bacillus species [35, 36] were the independent variables associated with dental caries of primary School children.

### Pooled effect size

Based on the pooled analysis of five studies, it was found that primary school children with a high intake of sweets are 2.71 times more likely to experience dental caries compared to those who consume fewer sweets (odds ratio [OR] = 2.71, 95% confidence interval [CI]: 1.968–3.451). Additionally, having a poor habit of tooth cleaning was significantly associated with dental caries. Students with poor tooth cleaning habits had 2.46 times higher odds of experiencing dental caries compared to those with good tooth cleaning habits (OR = 2.46; 95% CI: 2.761–5.045). Furthermore, being in grade levels 1–4 was significantly linked with dental caries. Students in these grade levels had 2.46 times higher odds of experiencing dental caries compared to those in higher grade levels (OR = 2.46; 95% CI: 1.523–3.397).

Having a history of toothache was also associated with dental caries. Students with a history of toothache were 2.99 times more likely to have dental caries compared to their counterparts (OR = 2.99; 95% CI: 2.679–3.314). Moreover, students who did not use toothpaste had 1.42 times higher odds of having dental caries compared to those who used toothpaste (OR = 1.42; 95% CI: -1.278-4.109). Furthermore, students with dental caries had 5.51 times higher odds of experiencing a reduction in their previous year’s academic score compared to those without dental caries (OR = 5.51; 95% CI: 1.952–9.066). Additionally, students with a significant microbial load had 3.82 times higher odds of having dental caries compared to those without a significant microbial load (OR = 3.82, CI: 3.439–4.192). Lastly, students with acidic bacillary pH on their teeth had 2.42 times higher odds of having dental caries compared to their counterparts (OR = 2.42, CI: 1.494–3.335). (Table 4).

**Table 4:** The pooled odds ratios of factors associated with dental caries among primary school children in Ethiopia

Factor Variables	Odds Ratio (95% CI) (Random effect model)	I 2(%)	*P*-value
High intake of sweets	2.71(1.968–3.451)	85.21	**0.0000**
A poor habit of tooth clean	3.90 (2.761–5.045)	96.15	**0.0000**
Grade 1–4	2.46 (1.523–3.397)	86.72	**0.0000**
Hx of Toothache	2.99 (2.679–3.314)	0.00	**0.0000**
Absence of toothpaste use	1.42(-1.278-4.109)	81.37	**0.3029**
Reduction of the previous year’s academic	5.51(1.952–9.066)	95.12	**0.0024**
Significant microbial load	3.82 (3.439–4.192)	0.00	**0.0000**
Acid bacillary ph	2.42(1.494–3.335)	82.74	**0.0000**

## Discussion

Presently, there is a rise in dental caries prevalence linked with the growth of emerging economies. Conversely, developed countries are experiencing a decline due to enhanced oral hygiene practices and the implementation of community-level intervention programs. However, the escalation in dental caries burden is likely associated with deficiencies in effective oral healthcare systems. These systems often prioritize curative care over the regular implementation of community oral health promotion initiatives [37, 38].

This systematic review and meta-analysis compiled evidence on the prevalence and factors associated with dental caries among primary school children in Ethiopia in the year 2024.

The pooled prevalence of dental caries among primary school children in Ethiopia was found to be 35%.

Based on the findings, the prevalence of dental caries identified in our current investigation exceeded that observed in Bahir Dar (21.8%) [21], and in Gonder 23.64% [39]. And in line with studies conducted in Gondar (41.5%) [23]. Furthermore, our discovery exhibited a lower rate compared to research carried out in Alemketema North Shiwa 46.9% [27], Addis Ababa (74%) [11], and Finote Selam (48.5%) [24] Eritrea (78%) [11], India (59%) [40], Qatar (85%) [41] Najran, Saudi Arabia (71.5%), Tamil Nadu (63.9%), and Brazil (55.5%) [11, 42, 43]. The variance in findings could potentially be attributed to differences in sample sizes and socio-demographic factors such as age categories and gender distribution.

The consumption of sweet foods showed a significant association with dental caries (AOR = 2.71; 95% CI: 1.968–3.451; *p* = 0.0000). This finding aligns with previous studies indicating that sugar intake is a key predictor of caries in Debre Birhane, harer, Addis Ababa, and Finote Selam [24, 34, 35, 44]. This agreement in results could be attributed to the subsequent activity of cariogenic bacteria, which produce abundant acid through the fermentation of sugar in sweet foods, thereby increasing enamel exposure to decay [45, 46].

In this study, there was a significant association between poor teeth cleaning habits and dental caries (AOR = 3.9; 95% CI: 2.761–5.04); *p* = 0.000). Participants who did not practice daily teeth cleaning had a higher prevalence of dental caries (35%). It’s well-known that cleaning teeth helps remove food debris from the mouth, depriving Lactobacillus spp. and other cariogenic bacteria of the nutrients and time needed for growth [24, 45].

There was a significant association between children’s grade level and dental caries, with 1st cycle (grades 1–4) students found to be 2.46 times higher risk compared to greater than grade five students. This study indicates that as grade level increases, the likelihood of dental caries decreases. This result is consistent with a previous report from Bahir Dar, which also found a two-fold higher prevalence of dental caries among 1st cycle students. The alignment with these findings may be attributed to the increased exposure to toothpaste among 2nd cycle students.

This study revealed that children experiencing dental aches were three times more likely to have dental caries. Similar results were found in Bahir Dar, where children with dental aches were 6.3 times more likely to have dental caries, and in a study conducted in Aksum, where children with dental pain were 1.8 times more likely to have dental caries. Similar studies were conducted in Addis Ababa, Sudan [47, 48] This correlation could be attributed to the possibility that dental aches are linked to poor tooth-cleaning habits and frequent exposure to sugary drinks and foods.

In this study, children who did not use toothpaste were significantly associated with caries development (AOR = 1.42; 95% CI: -1.278-4.109; *p* = 0.3029). Dental caries was lower among those who cleaned their teeth with a toothbrush and toothpaste compared to those who did not. This is due to a lack of knowledge regarding proper toothbrush usage among users, which is consistent with findings from a study in Addis Ababa where children experienced gum bleeding while brushing. Despite the availability of fluoride toothpaste in the study area, the knowledge and utilization of fluoride might have implications for the caries burden among both users and non-users of toothpaste.

The majority of children with dental caries achieved lower scores in their previous year’s cumulative assessments (AOR = 5.51; 95% CI: 1.952–9.066; *p* = 0.0024). This could be attributed to the effects of caries and associated infections, which can cause pain and discomfort, resulting in decreased attention and school absenteeism. This, in turn, directly impacts children’s academic performance and productivity.

Significant Lactobacillus microbial load is significantly associated with dental caries [(AOR = 3.82, 95% CI: 3.439–4.192 *P* = 0.000)]. This could be attributed to the effects of caries and associated infections, which was due to high bacterial counts are, in any case, an indicator of a high caries risk, i.e. latent risk of developing caries.

### Implications of the study

The findings of the systematic review and meta-analysis highlight the urgent need for comprehensive, multi-sectorial approaches to address the escalating burden of dental caries among primary school children in Ethiopia. By targeting modifiable risk factors, promoting oral hygiene practices, and strengthening healthcare systems, it is possible to mitigate the impact of dental caries and improve overall oral health outcomes in this population.

### Strengths and limitations of the study

Comprehensive search strategy across multiple databases, ensuring a comprehensive retrieval of relevant studies, Adherence to PRISMA guidelines in the study selection process, minimizing bias and ensuring transparency, Risk of bias assessment using the Hoy 2012 tool, enhancing the reliability of included studies and Statistical analysis including meta-analysis, meta-regression, and sensitivity analysis, providing robust and reliable synthesis of data.

There is a possibility of biases in the study, including inaccuracies in selecting study participants, small sample sizes in certain studies, limitations in data collection and analysis, and selective reporting of results in the included studies. These biases could potentially influence the findings of the meta-analysis. Additionally, there may be variations in study quality among the included studies, which could impact the overall quality of evidence.

### Conclusion and recommendation

In this study, the prevalence of dental caries was higher and a common public health problem among school children. High intake of sweets, grade level 1–4, a poor habit of tooth cleaning, toothache, reduction of previous year academic year score, absence of toothpaste use, and significant microbial load of slivery lacto bacillus species were the independent variables associated with dental caries of primary school children’s.

Our findings indicate that the Ethiopian Federal Ministry of Health should prioritize efforts to enhance the oral healthcare system and implement community-level intervention programs with greater emphasis, Immediate restorative dental services should be made available for individuals with decayed teeth, and also Communities should establish a system for regular dental check-ups every six months, and adherence to this schedule should be encouraged.

## Data Availability

All data included in systematic review and Meta-analysis are available in the main manuscript.
