# Correction: Prevalence of dental caries and associated factors among primary school children in Ethiopia: systematic review and meta-analysis

**DOI:** 10.1186/s12903-024-04645-4

**Published:** 2024-07-26

**Authors:** Amlaku Nigusie Yirsaw, Eyob Ketema Bogale, Mitiku Tefera, Mahider Awoke Belay, Ayenew Takele Alemu, Solomon Ketema Bogale, Eyob Getachew, Getnet Alemu Andarge, Kedir Seid, Gebeyehu Lakew

**Affiliations:** 1 Health Promotion and Communication Department, School of public health, College of medicine and health sciences, Gondar University, Gondar, Ethiopia; 2 Health Promotion and Behavioral science department, school of public health, College of medicine and health science, Bahir Dar University, Bahir Dar, Ethiopia; 3 Department of Midwifery, School of Nursing and Midwifery, Debre Birhan University, Asrat Woldeyes Health Science Campus, Debre Birhan, Ethiopia; 4 Department of Public Health, College of Medicine and Health Science, Injibara University, Injibara, Ethiopia; 5 Department of Nutrition, Antsokiya Gemza wereda Health Office, Mekoy, MekoyNorth East Ethiopia; 6 Bati Primary Hospital, Oromo Special Zone, Bati, North Central Ethiopia

**Correction to: BMC Oral Health (2024) 24:774**

10.1186/s12903-024-04555-5.

In this article [1], the author name Amlaku Nigusie Yirsaw was incorrectly written as Amalku Nigusie Yirsaw. The author name was corrected in this version.
